# Robots at your doorstep: acceptance of near-future technologies for automated parcel delivery

**DOI:** 10.1038/s41598-023-45371-1

**Published:** 2023-10-29

**Authors:** Maher Said, Spencer Aeschliman, Amanda Stathopoulos

**Affiliations:** 1 Department of Civil and Environmental Engineering, Northwestern University, A308 Technological Institute, 2145 Sheridan Road, Evanston, IL 60208 USA; 2 Department of Civil and Environmental Engineering, Northwestern University, A312 Technological Institute, 2145 Sheridan Road, Evanston, IL 60208 USA

**Keywords:** Statistics, Civil engineering

## Abstract

The logistics and delivery industry is undergoing a technology-driven transformation, with robotics, drones, and autonomous vehicles expected to play a key role in meeting the growing challenges of last-mile delivery. To understand the public acceptability of automated parcel delivery options, this U.S. study explores customer preferences for four innovations: autonomous vehicles, aerial drones, sidewalk robots, and bipedal robots. We use an Integrated Nested Choice and Correlated Latent Variable (INCLV) model to reveal substitution effects among automated delivery modes in a sample of U.S. respondents. The study finds that acceptance of automated delivery modes is strongly tied to shipment price and time, underscoring the importance of careful planning and incentives to maximize the trialability of innovative logistics options. Older individuals and those with concerns about package handling exhibit a lower preference for automated modes, while individuals with higher education and technology affinity exhibit greater acceptance. These findings provide valuable insights for logistics companies and retailers looking to introduce automation technologies in their last-mile delivery operations, emphasizing the need to tailor marketing and communication strategies to meet customer preferences. Additionally, providing information about appropriate package handling by automated technologies may alleviate concerns and increase the acceptance of these modes among all customer groups.

## Introduction

The last-mile logistics of freight distribution is a critical and challenging aspect of the supply chain. It is often the least efficient and most expensive stage^1–3^, accounting for up to 28% of delivery transportation costs, and is a considerable source of congestion and other externalities, such as pollution and noise^2–5^. These inefficiencies and negative impacts on citizen well-being make improving last-mile logistics a priority for businesses and policymakers alike^2^. Last-mile delivery challenges are likely to become more pressing due to factors such as increased e-commerce, rising consumer expectations, and the growth of ride-hailing competing for scarce curbspace^6^. Increased e-commerce and expectations for speed by consumers have been tied to the growing volume of parcels, smaller shipment sizes, and higher frequency of delivery trips^7^. Additionally, the rapid proliferation of ridehailing services presents a series of novel challenges to the effective management of curbside operations, including reduced available space for loading and parking for conventional urban delivery vehicles^8–10^. The global COVID-19 pandemic, the transformation of work, and a corresponding surge in e-commerce and delivery demands^11,12^ have also brought to light critical vulnerabilities in the supply chain, particularly related to cascading disruptions, lasting reliance on residential home-deliveries, and labor shortages^13,14^.

To address these challenges, firms such as Amazon, Walmart, Einride, Eliport, and UPS are exploring the deployment of autonomous freight delivery options^15,16^. The market size for automated delivery technologies, including autonomous vehicles (AVs), drones, and robots, is projected to reach $665 billion (about $2,000 per person in the US) by 2030, representing up to 20% of the package delivery industry^17^. These technologies offer numerous benefits, including greater efficiency, safety, and sustainability, and can help reduce human error. Smaller-scale automation technologies, such as drones and delivery robots, can offer more efficient, safer, cheaper, and sustainable solutions than traditional truck deliveries by, for example, bypassing congested streets and curbsides^15,16^. Yet, the shift towards automation can be fraught with negative effects on employment, safety, and security, and open questions about shipping performance, operational needs, and regulatory support^18^. However, by combining various automated delivery technologies, such as launching drones from autonomous vehicles, it is possible to mitigate these drawbacks and enhance efficiency compared to single technology operations^15,16^. Combined systems can address obstacles for ground delivery in urban environments and expand service in suburban and rural areas, where higher delivery costs are a significant challenge^19–21^. Thus, the (combined) deployment of robots, autonomous vehicles, and drones holds promise for addressing reliance on human drivers, managing curbside challenges, meeting growing demand and customer expectations, and preparing for future disruptions^22–24^.

The materialization of these benefits, however, is dependent on the adoption and public acceptability of these technologies^25^. While most acceptability research to date has focused on self-driving vehicle use among passengers^19–21,26,27^, limited insight exists on customer attitudes toward near-future automated delivery modes^25,28–33^. Both delivery performance attributes and attitudes toward automated services will play a role in their acceptance. Existing studies find that acceptance is linked to the perceived usefulness, convenience, and flexibility of automated parcel delivery^28,31–36^, all constructs related to cost and delivery speed. Studies also identify several adoption barriers, namely concerns over package handling, security, and privacy, as well as a lack of trust and familiarity^25,28–33^. The role of environmental concerns is not yet clear. Research is still needed to determine the impact of automation on supply chain sustainability. For example, recent simulation work suggests that while drones offer some energy efficiency improvements over traditional delivery, their per-day energy consumption would be comparable to battery electric vehicles on normal days and as high as diesel trucks on windy days^37^. Other work suggests that drone delivery leads to lowered CO_2_ emissions for logistics^38^. Additionally, the link between customers' perceptions of the environmental impact of delivery automation and their acceptance of these technologies remains ambiguous^39^. A general takeaway is that the acceptability of automated delivery options depends on a complex set of attitudinal, demographic, and market-based factors. A current challenge is that studies generally focus on a single technology (e.g. Figliozzi and Jennings^40^ and Hwang et al.^41^), while in reality customers will likely be faced with a portfolio of innovative options, and make trade-offs between several delivery attributes at once. A notable exception is Polydoropoulou et al.^42^, who study a multi-option decision context in a choice experiment in Greece, finding that respondents were generally unwilling to opt for innovative delivery modes over traditional ones due to cost, lack of familiarity, and infrastructure barriers. More research is needed to understand the relative importance of these different factors and how they interact, especially in light of multiple competing or complementary automation technologies, and different urban delivery contexts.

This paper aims to understand the potential acceptability of near-future automated delivery technologies among U.S. customers. The paper makes three main contributions to the literature. *First*, we study the adoption likelihood of a portfolio of multiple innovative delivery automation technologies in tandem. We design a Bayesian efficient choice experiment including traditional delivery along with four automated delivery innovations: (a) autonomous vehicles, (b) drones, (c) sidewalk robots, and (d) bipedal robots. This multi-technology setting enables us to examine patterns in customer acceptability and relationships among similar technologies. *Second*, we examine the role of shipping attributes, shipped item type, and socio-demographic and behavioral variables, thereby revealing the role of personal and shipping characteristics on the preference over future technologies. *Third*, we explore the impact of attitudes on delivery mode preferences. Specifically, we study the role of packaging preferences, environmental awareness, and affinity towards technology on the acceptability of automated delivery technologies.

Using an Integrated Nested Choice and Correlated Latent Variable (INCLV) model, we account simultaneously for dependence across alternative technologies and for correlated attitudinal latent variables, uncovering critical factors that influence customer acceptance of automated parcel delivery innovation. The model reveals the importance of attributes like shipment price, time performance, customer-specific attributes like age and education, and attitudes such as concerns about package handling and affinity towards technology. Scenario simulation is applied to examine business strategies and contextual events on the preference for automation. Our findings provide valuable insight and recommendations for analysts, policymakers, and practitioners, including retailers and couriers, seeking to successfully introduce these technologies to the market while mitigating their negative impacts on customers, society, and the environment.

## Results

**Figure 1:**
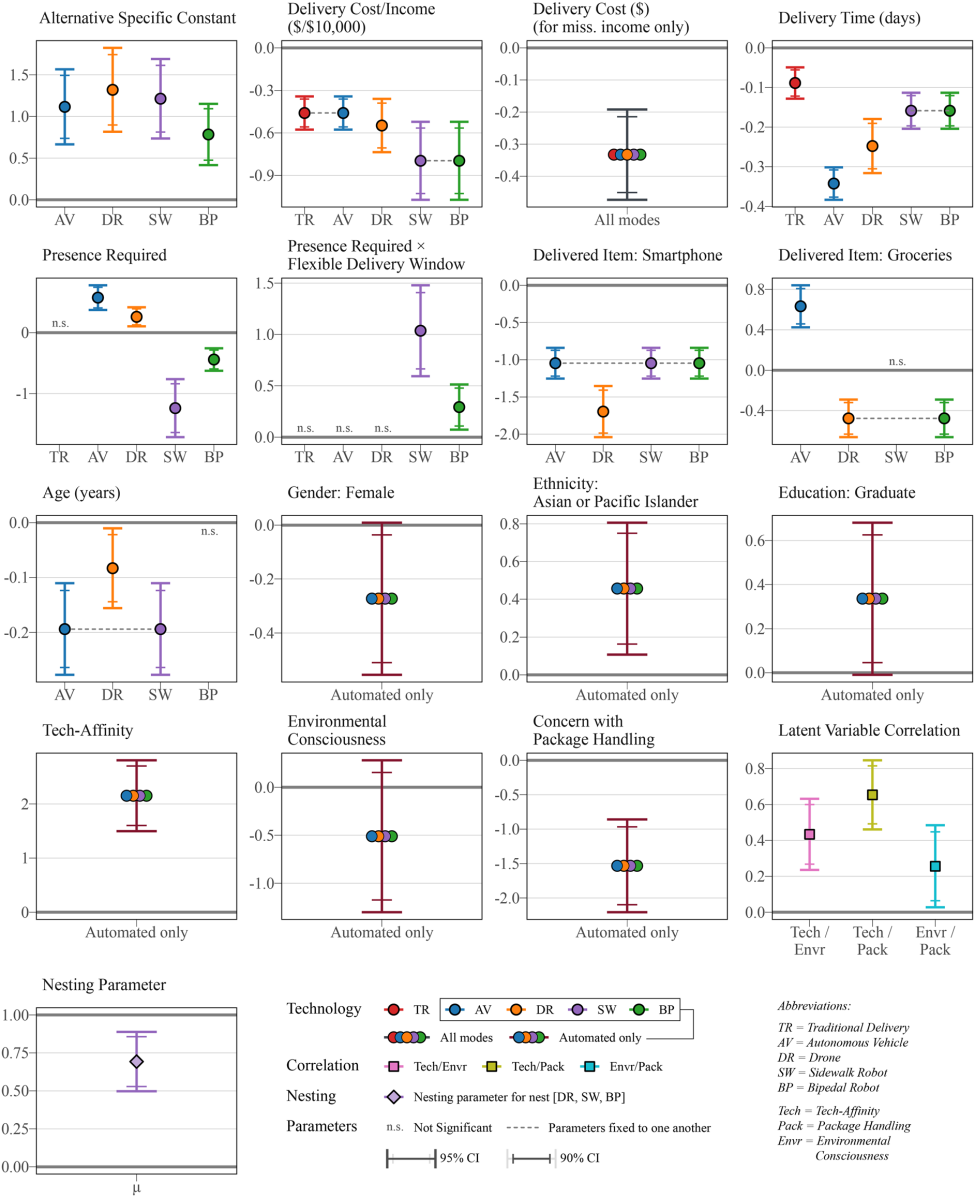
Resulting coefficients of INCLV model for novel delivery technologies.

Using choice experiment data from 692 U.S. respondents, we construct an extensive Integrated Nested Choice and Correlated Latent Variable (INCLV) model to assess preferences towards four novel parcel delivery technologies and nuances in decision-making across these technologies. In addition to typical mode- and user-specific attributes (such as cost and income respectively), the model also incorporates relevant latent attitudes based on the literature: *affinity towards technology*, *environmental consciousness,* as well as a novel construct *concerns with package handling*. Details on data collection, survey design, methodology, and modeling are provided in the “Methods” section. The results for the estimated model are presented in Fig. 1 (see Supplementary Tables S1 and S2 for model statistics and model results in numeric form).

**Figure 2:**
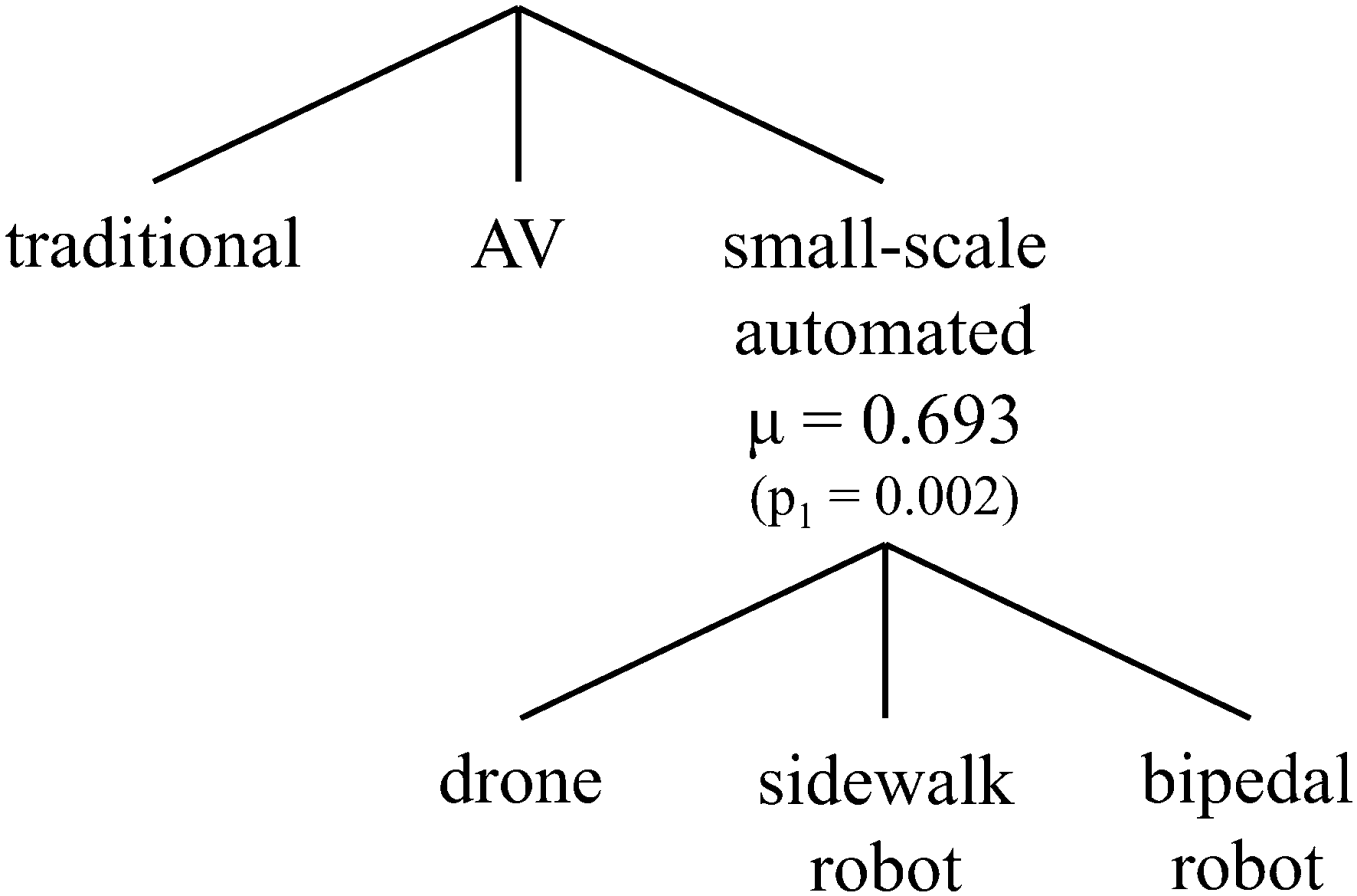
Nesting structure of selected INCLV model.

The best-performing nesting structure is provided in Fig. 2. To maintain parsimony given model complexity, coefficients with similar values and overlapping confidence intervals are selectively fixed to the same value, excluding alternative specific constants. The most important findings are discussed in the next sub-sections, followed by scenario testing.

### Cost and time performance are strongly related to automation-avoidance

**Figure 3:**
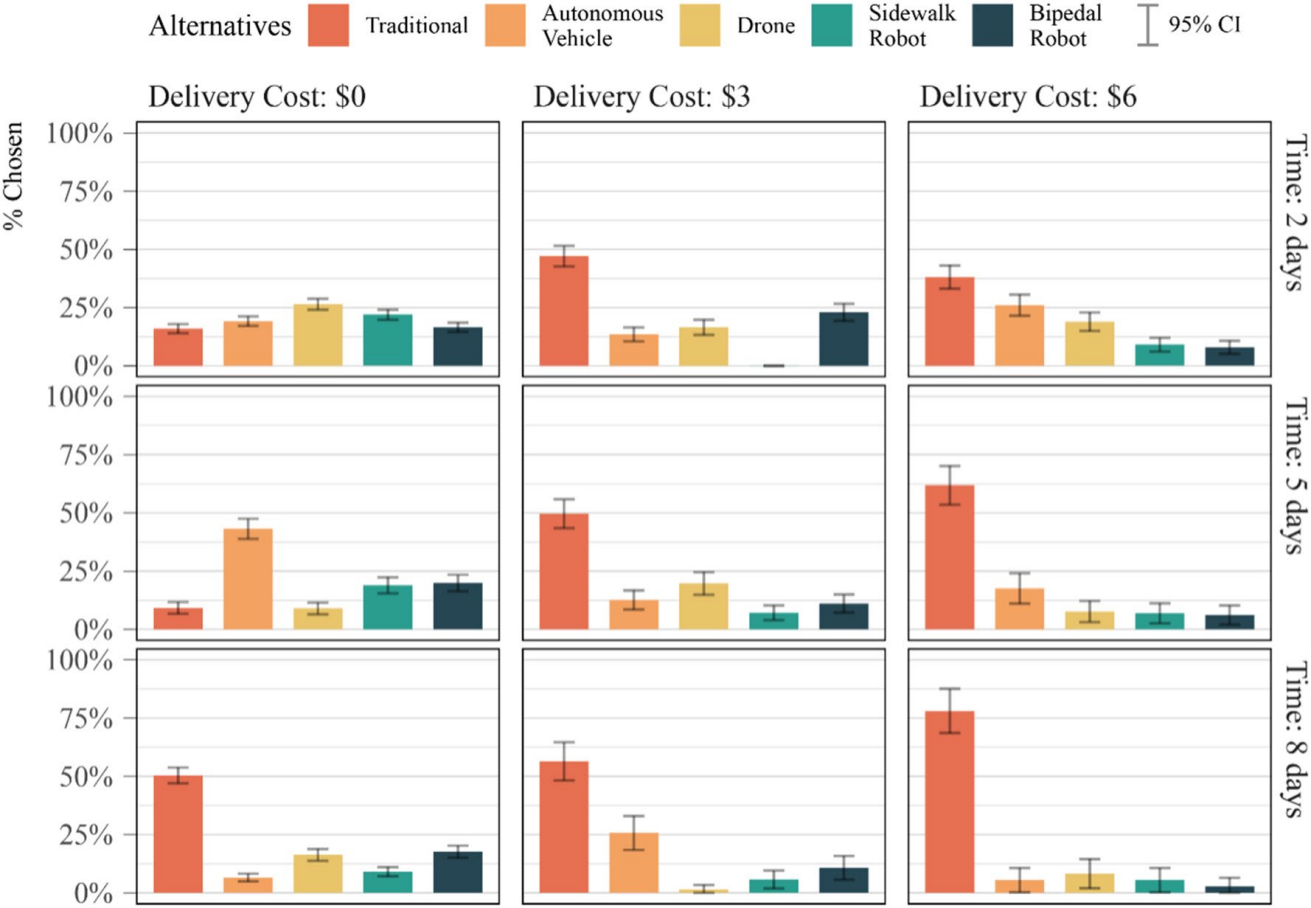
Delivery mode choice distribution as a function of delivery cost and delivery time across all choice scenarios. 95% confidence intervals are presented for each bar. Deliveries get more expensive and take longer from left to right and top to bottom, respectively.

Our study highlights an interesting relationship between novel technology acceptability and the relationship with both delivery cost and time performance, as shown in Fig. 3. When customers are offered high-performing shipping scenarios, such as free 2-day deliveries, preferences across alternatives (in aggregate) become more evenly distributed, with some inherent preference for drones. However, as cost and time increase, choices quickly skew towards traditional delivery modes, especially as a function of cost. This suggests a growing willingness to give up on traditional truck delivery and experiment with novel delivery automation options in a frictionless idealized cost and time performance scenario.

### People exhibit distinct preferences for AV versus small-scale automated options

We find that people have a generally positive intrinsic preference towards transformative automation technologies compared to traditional truck delivery, as revealed by the significant constants. As seen in other works, exuberance and novelty-seeking surrounding new technologies are not uncommon^41,43^. Among the new delivery options, the lower constant for *bipedal robots* indicates a lower innate preference for that mode, likely because it represents the most jarring leap in technology among the options. The nested logit formulation reveals a heightened substitution effect among the smaller automated delivery methods, being *drone*, *sidewalk robot,* and *bipedal robot.* The corresponding nesting parameter, $\mu$, is significantly different than 1.0 (p_1_ = 0.002) with a value of 0.693, indicating that these smaller automation options are closer substitutes with some separation from the *autonomous vehicles.* Following extensive testing of alternative nesting hypotheses (Supplementary Fig. S1 compares), there is no solid evidence of another bundling of options, suggesting that these different automated options are mostly regarded as a similar group. This is a major takeaway from this work, pointing to the fact that people mentally bundle drone and robotic options together. In turn, this guides practical considerations as the success of one automated technology is expected to positively impact the performance of other technologies in the same bundle. This insight is only possible due to the experimental design presenting several automated options together for joint evaluation.

The important impact of drop-off location for each delivery mode could be captured through different nesting structures. However, as shown in the selection process for the nesting structure (see Supplementary Fig. S1), any nesting based on drop-off location is reduced to a simple multinomial logit (MNL) model, implying there are no substitution effects among modes sharing similar delivery locations, be that in terms of placement or security. We note that a future experiment with more details on future drop-off technologies like trunk-delivery or residential lockers may yield a different nesting result. Even so, the effect of drop-off location may still be captured implicitly via other parameters present in the model as discussed in the forthcoming section discussing the ‘last steps’ of delivery.

### The critical role of value-of-time for acceptance

Cost is a strong driver for adoption across all modes, with all delivery cost/income coefficients being significant (p < 0.001). Notably, the cost sensitivity towards *traditional truck* delivery and delivery through an *autonomous vehicle* (both vehicle-based) is found to be roughly equal, leading us to fix the parameters to one another in the model specification. The cost sensitivity is shown to be the lowest for the more familiar traditional and AV alternatives. On the other hand, respondents are, on average, 1.73 times more sensitive to delivery costs through technologies such as *sidewalk robots* and *bipedal robots*.

Similarly, all time coefficients are highly significant (p < 0.001). Interestingly, time sensitivity has autonomous vehicle sensitivity breaking away from traditional vehicles, suggesting that customers have a higher performance expectation for AVs. Moreover, respondents are roughly 2–4 times more sensitive to increased delivery times when the delivery mode is non-traditional compared to the current status quo. These results highlight that delivery *convenience* must be prioritized in the large-scale rollout of automated delivery modes. This is especially true for deliveries performed through *automated vehicles* or *drones*, where people exhibit higher sensitivities towards delivery performance.

The resulting value-of-time (VOT) for receiving deliveries is equal to $1.42/day for traditional deliveries and $2.92/day for automated deliveries. The values for traditional delivery are in line with current delivery costs: using 103 data points manually collected on Amazon for a variety of delivery items, the slope for delivery prices within the sample is equal to $1.40/day. We caution the reader from interpreting the increased VOT for automated deliveries as respondents being willing to pay higher prices for automated deliveries, but rather as a need to be compensated in terms of improved delivery performance to accept the risk and uncertainty of automated technologies (in line with Figs. 1 and 3).

### The ‘last steps’ of drone and robot delivery: presence requirement attribute

During the pandemic, many customers gained experience from curbside pickup and other last-mile delivery innovations^44^. Robots and drones present new logistical ‘last steps’ challenges when delivering items to customer homes, including limited doorstep accessibility and leaving items unattended. Our work provides evidence that requiring the presence of a recipient plays different roles depending on the delivery technology. In the case of traditional delivery, this requirement—or the lack thereof—has no impact on alternative selection, having an insignificant coefficient. On the other hand, for *autonomous vehicle* and *drone* deliveries, the presence requirement is significant and positive. Given that the delivery locations for these two modes are not particularly secure, the presence requirement is likely perceived as a guarantee of successful delivery here rather than an inconvenience. The least secure of these two modes, *autonomous vehicle*, has a higher coefficient of 0.575 (p < 0.001) compared to 0.260 (p = 0.001) for *drones*. Among respondents offering open-ended feedback, 78% (31 out of 40) expressed concerns regarding shipping modes that are incapable of doorstep or secure delivery.

**Figure 4:**
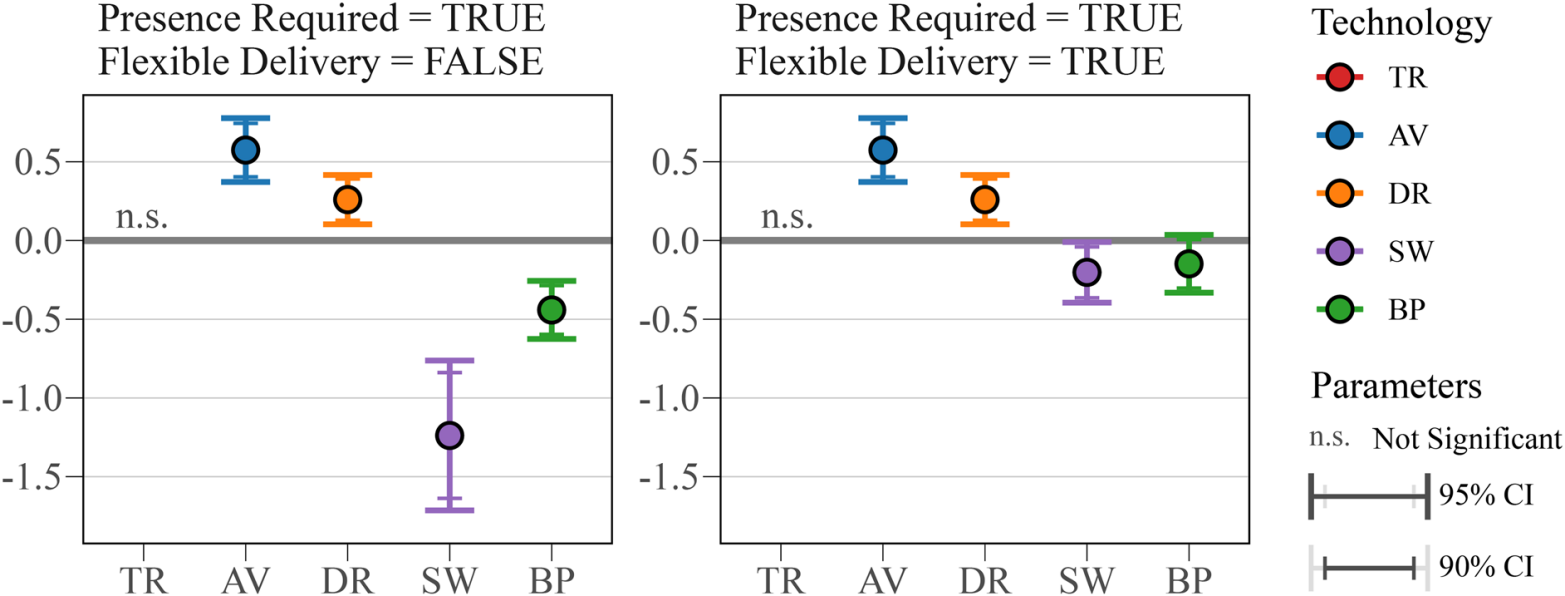
Interacted coefficient for presence requirement with and without flexible delivery.

Instead, for technologies that provide more secure local deliveries, namely *sidewalk* and *bipedal robots*, the presence requirement has a negative impact. When evaluating delivery robots, customers view the presence requirement as an inconvenience. The impact for *sidewalk robot* is especially large, with a value of − 1.24 (p < 0.001) compared to − 0.441 (p < 0.001) for *bipedal robot*. Further insight is revealed by considering attribute interactions. This negative impact of requiring presence is notably reduced when accompanied by the option of a flexible delivery window. As illustrated in Fig. 4, this effect is reduced sixfold for the *sidewalk robot* (p = 0.040) and rendered insignificant for *bipedal robot* (p = 0.114). In other words, for the delivery robots, if deliveries have flexible timing that can be suited to people’s schedules, customers feel less inconvenienced by needing to be available for delivery, if at all.

### The parcel content matters: food versus technology-based shipments

Earlier research rarely considered the specific shipping item in tandem with technology-type in determining delivery acceptability (e.g. Hwang et al.^41^). We examine three types of shipment contexts in the choice experiment (see Supplementary Table S3 showing the experimental design). A commonly shipped item is used as a reference in the modeling, namely a book, which is cheap, light, and neither fragile nor perishable. In the case of an expensive and fragile item such as a smartphone being delivered, the coefficients for novel technologies are negative and significant (p < 0.001). This finding reveals that trust towards automated delivery overall is lower for fragile shipping items like smartphones. Adding these coefficients to the alternative specific constants for automated technologies renders them insignificant at the 90% level of confidence, nullifying any innate interest in these technologies. In the case of drones, an extra dimension is revealed through the larger magnitude (− 1.70 vs. − 1.05), where the risk of damage due to aerial delivery is likely considered. Such concerns have been voiced in a report by the US Postal Office^45^.

As for groceries, the perception of different modes is more divisive. The coefficients suggest that, at least during the initial stages of adoption, self-driving cars would be the most acceptable option for delivering groceries compared to other delivery technologies. We speculate that the more visceral relationship people have with food handling and delivery, as well as their growing experience with on-demand food delivery apps during the pandemic has contributed to this finding^29^. Moreover, customers likely have concerns about the capability of smaller-scale technology like drones and robots to safely carry and deliver bulky or perishable produce. Grocery delivery is, therefore, best served by the more familiar option in the automation portfolio. Seven respondents (out of 40 who provided open-ended comments) highlighted their concerns with using automated delivery for more valuable, expensive, or perishable items. Automated technologies are, at least initially, better catered to the delivery of cheaper non-perishable items.

### Individual differences in automation acceptance

The model search process revealed that most socio-demographic factors have a technology-agnostic impact on acceptance. That is, outside of age, all sociodemographic and attitudinal attributes impact adoption likelihood equally across the four automated options in comparison to traditional truck delivery. Looking at *age*, preference for *automated vehicle*, *drone,* and *sidewalk robot*, compared to traditional delivery, is lower among older individuals. This negative perception is stronger for *automated vehicle* and *sidewalk robot* (β = − 0.194) compared to *drone* (β = − 0.0832). In the case of *bipedal robots*, however, the perception is not significantly different than that of traditional delivery across age groups. Whereas it may seem natural to expect a dislike of this option among older cohorts due to the larger technological leap, we hypothesize that secure doorstep delivery is a large driver in mode preference for older respondents.

While only significant at a 90% level of confidence, the model indicates that preference for automated delivery technologies is lower among women (p = 0.058). This finding is in line with the literature, with men—all other things being equal—typically showing a higher preference for novel technologies^29,46^. Although the data includes 14 respondents (2.0% of 692) who identified their gender as non-binary, alternative model specifications using *male* or *female* as the base gender have yielded insignificant coefficients for *non-binary genders* in both cases. Therefore, with only a few observations, the effect of *non-binary gender* is inconclusive. The model also reveals—at a 90% level of confidence—a higher preference for novel delivery options among individuals with graduate-level education (p = 0.057). In terms of ethnicity, a higher acceptance of automation is observed among *Asian* and *Pacific Islander* respondents.

### Acceptance is adversely shaped by key attitudes: package handling and technology affinity

Individuals with a higher affinity towards technology are more likely to opt for automated delivery options. This indicates that promotional efforts should be directed at individuals who engage with technology in other areas. On the other hand, we did not find any support for environmental concerns driving the demand for automated delivery alternatives.

Finally, respondents who are more concerned with how their deliveries are handled are less likely to adopt automated technologies, suggesting a lack of trust in these technologies and a preference for tried-and-tested traditional modes. The findings concerning privacy and package handling are in line with results previously seen in the literature^32,36,47^. Interestingly, there is a positive correlation between the latent variable estimates for *technology affinity* and *concern with package handling.* At first glance, this may appear counterintuitive. However, the strong associations suggest that concern towards package handling is closely related to technological proficiency, encompassing aspects such as shipment tracking and security. Despite this, the impact on acceptability is contrary, indicating that non-technology aspects of parcel delivery handling are a key driver of hesitancy. This invites further research on building trust and adoption comfort by carefully managing parcel-handling concerns.

There was no evidence for specific pandemic impacts on responses. Several questions about the impact of the COVID-19 pandemic have been tested and found to be consistently insignificant in the model. These factors include the impact of the pandemic on household employment, income, health, opinions, and life overall. This underscores the generalizability of the model and results beyond the pandemic.

### Simulations of future delivery scenarios

**Figure 5:**
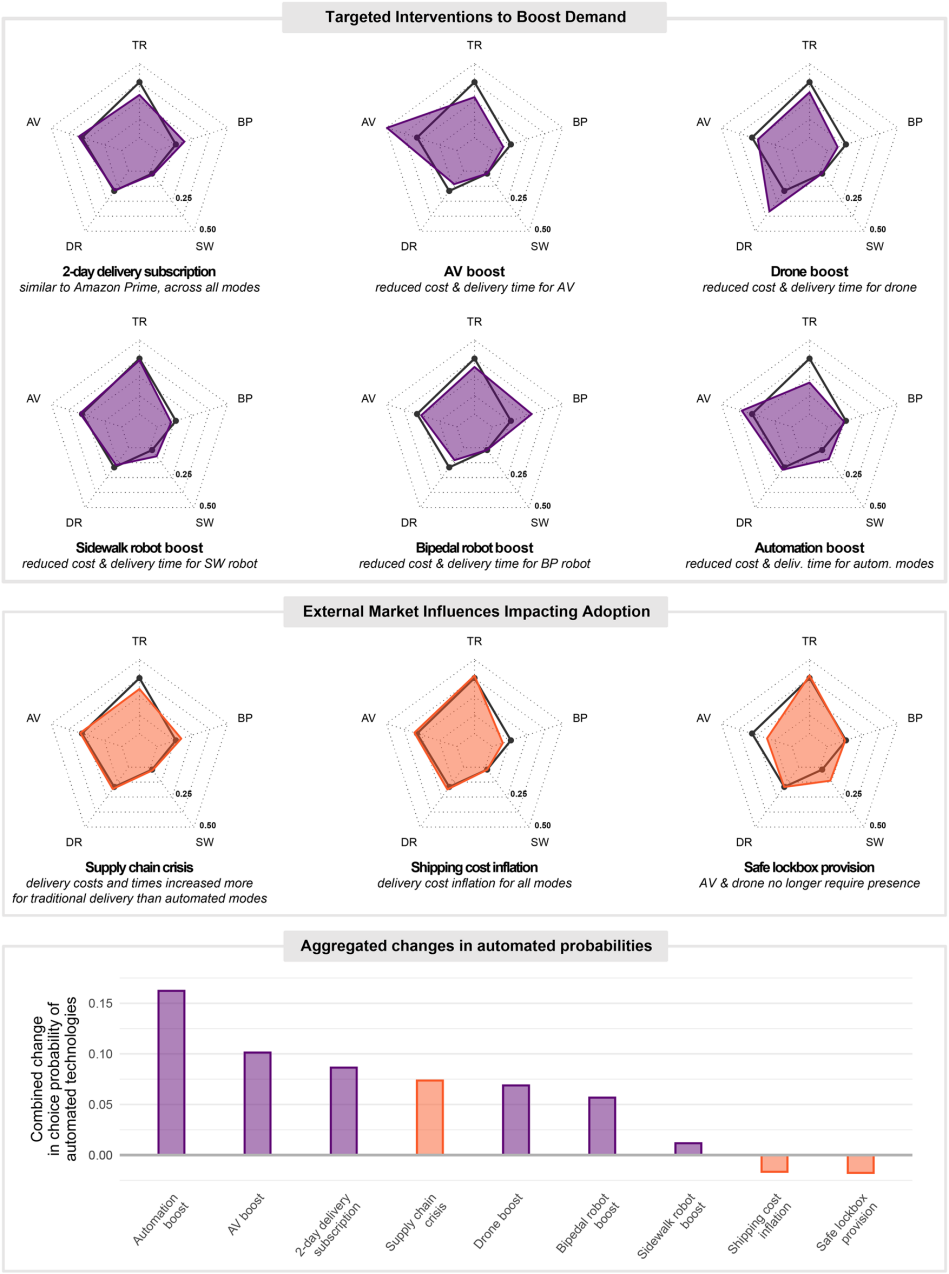
Choice probabilities for nine policy scenarios. The black polygon in the spider plots represents the baseline market-share. The top six plots (purple) illustrate targeted firm strategies for promoting specific technologies or service performance by decreasing delivery times and costs. The middle three plots (orange) test broader market and infrastructure scenarios. The bottom bar plot ranks the cumulative automation market-share for each scenario.

To delve deeper into the model insights and gain a more comprehensive understanding of the future landscape of the delivery industry and public acceptance, we employed the final INCLV model to investigate a variety of scenarios. Our scenario simulations (Fig. 5) encompass a wide range of possibilities, divided into two groups: (a) six targeted firm interventions and (b) three external market influences (see Supplementary Table S4 for details on scenario designs). To simulate mode shares for each scenario, we employed the method of Sample Enumeration, outlined in Train^48^.

We first define a baseline scenario to compare against the targeted firm intervention and external market influence scenarios. The market shares for the baseline are shown as the black line in Fig. 5 and are intended to represent a near future with a fairly level playing field in terms of delivery costs and times across delivery modes. The attribute values for the baseline were selected based on averages obtained from a pilot investigation (n ~ 110 data points) of delivery costs and times for a wide variety of comparable items that the authors carried out on Amazon (not Prime) in April 2020. The baseline helps contextualize these policy scenarios by representing the “do nothing” market share. These baseline values are shown in the first column of Supplementary Table S4.

We then define scenarios by assigning values to the four delivery attributes: delivery time, delivery cost, presence required, and flexible delivery window, according to the given policy scenario. These four attributes are the only variables that change across scenarios. To estimate the hypothetical mode shares in each scenario, we take the original sample and assign the estimated Beta coefficients and latent variable coefficient values to each individual, while maintaining the same observed personal characteristics. Choice probabilities are then recomputed for every individual in the sample, using the same item types and delivery locations in the original choice scenarios, plus the scenario-assigned delivery attributes. The averages of these choice probabilities constitute the mode shares for the given scenario shown in shaded areas in Fig. 5.

The targeted firm intervention scenarios (“boost” scenarios) involve specific measures to support each delivery technology. These include promotional low prices and short delivery windows for individual technologies, as well as an Amazon Prime-inspired scenario where all delivery options are offered at a subscription price with 2-day delivery windows. Additionally, a general “automation boost” scenario considers performance improvements across all automated options. On the other hand, the three “external market influence” scenarios account for indirect market shifts that may impact the adoption of these technologies. This approach allows us to examine the potential effects of both targeted interventions and broader market changes on the future landscape of the delivery industry. In the “boost” scenarios, delivery costs and times are decreased for the target mode relative to the baseline, and in the “external influence scenarios”, costs and times are altered along with other delivery attributes to reflect larger-scale external market influences.

Examining the “boost” scenarios for individual modes, we observe that AVs, drones, and bipedal robots exhibit significant responsiveness to lower costs and expedited delivery times. In contrast, sidewalk robot acceptance demonstrates limited responsiveness to these service enhancements. In the “automation boost” scenario, where automated modes offer flexible deliveries without requiring a presence, sidewalk robots experience a more substantial increase in choice probability as customers transition away from traditional truck delivery. Further insights are obtained by examining the external market scenarios. In these cases, sidewalk robots greatly benefit from the availability of *safe lockboxes*, resulting in a substantial shift of approximately 10% in choice probability from AVs. Interestingly, in the scenario of *shipping cost inflation*, where all modes experience delivery cost and time increases relative to the baseline (and more so for the traditional mode reflecting labor shortage issues), choice probabilities prove resilient to change, except for the declining probability of selecting bipedal robots, despite the price for traditional delivery increasing more markedly in this scenario. The lower bar plot panel of Fig. 5 shows that “boost” scenarios generally prove more successful in promoting overall automated delivery growth in our simulations, suggesting that they should be pursued concurrently with broader market and infrastructure changes.

Market disruption scenario analysis uncovers further non-uniformity in choice probability shifts across automated modes, extending beyond the nesting among drone and robot-based delivery. Sidewalk robots distinctly benefit from enhanced convenience in the form of flexible deliveries without the necessity of a customer’s presence. This is consistent with the strong negative marginal effect of the *presence required* variable and the positive marginal effect of the *flexible delivery window* variable (see Supplementary Table S2). These results also shed light on the dynamic interplay among automated delivery technologies. For example, in the “sidewalk robot boost” scenario, the majority of the additional mode share for sidewalk robots is derived from a reduction in the bipedal robot mode share. Conversely, in the “safe lockbox provision” scenario, sidewalk robots mainly gain additional mode share at the expense of autonomous vehicles. These observations highlight the complex dynamics between various delivery technologies and their adaptability to diverse situational contexts.

## Discussion

This study offers important insights into potential customer adoption of new last-mile delivery technologies, including automated vehicles, drones, and robots. As a general takeaway, we observe that the behavioral mechanisms for the acceptance of near-future technologies for automated parcel delivery are complex and multifaceted. Our study shows that customers do not evaluate innovative shipping options as independent alternatives. Instead, acceptance of small-scale automation alternatives is shaped by higher levels of substitution, suggesting that the drone and robot-based options are regarded as a similar group. Furthermore, we find that in terms of several choice determinants, the novel automated options are displaying some uniformity in preferences. This is true for customer-specific attributes such as gender, education, and attitudinal factors. Acceptance of these technologies can therefore be considered as a partially bundled behavior, where the adoption of one technology can extend to the adoption of other automated goods delivery options.

A notable deterrent to using automated delivery technologies is the concern about the potential mishandling of deliveries and packages by these modes. This is further evidenced by a reduced preference for automated delivery modes in the case of more expensive and fragile deliveries in comparison to other basic items. Alleviating these concerns through the dissemination of information and marketing regarding appropriate package handling by automated technologies provides an effective route for encouraging the acceptance of automated modes. Indeed, through simulations, we observe that a 10% decrease in concern towards package handling results in an 8.3% increase in the adoption of automated options.

We next offer takeaways in the policy and practice context. Looking into a future where logistics systems are increasingly automated, local governments and planning departments can gain insights from our study. Likewise, retailers and carriers contemplating a shift towards automation will gain understanding of how different automated delivery services are viewed by customers. Looking at retailers, logistics operators, and the courier industry, the study gives important insight into potential demand and how specific service and user-variables are connected to customers' interests. We find that automated delivery options are 2 to 4 times more sensitive to delivery times in comparison to traditional truck operations, highlighting the value attributed to speedy deliveries—and therefore convenience—for automated options. Notably, attitudinal motivations appear to be consistent across the four innovative technologies. At the same time, the scenario analysis reveals consumer differentiation between interest in automated modes given changing contexts. These findings suggest a need for careful communication about the effects of automation. It is known that many logistics decisions result from interactions between shippers and carriers^49^ and that the choice of carrier arrangement is crucial for companies^50^. Therefore, given the many changes that accompany automation of supply chains, it is key to also carefully coordinate the adoption of automated delivery innovations, and track performance changes. From the customer perspective, combined increases in shipment time and cost rapidly trigger a return to traditional delivery mode preferences. This necessitates that automated delivery rollout and incentives be carefully planned and coordinated to maximize the trialability of innovative logistics options to overcome consumer reservations.

From the perspective of city policymakers and regulators who are planning for future logistics innovations, our study provides complimentary insight into adoption patterns. Scenario simulations that mimic different logistics incentives show that AVs, drones, and bipedal robots are primed for substantial adoption should they be priced competitively with fast service. The adoption of sidewalk robots, on the other hand, is more heavily driven by convenient and secure delivery systems. This suggests that policymakers have an important role to play in facilitating adoption by supporting the development of infrastructure or regulations. At the same time, policymakers need to consider the interests of the general public in addition to the receivers of e-commerce. However, given how widespread direct-to-consumer shipments are, this is likely an important constituent share.

Our study has caveats that warrant consideration and invite further work. *First*, we frame our study from the perspective of customer acceptance. In practice, delivery automation is likely to be advanced by shippers and retailers and impacted by the response from delivery workers and the public. Therefore, the acceptance process will be shaped by several stakeholders and framed by firm strategies and regulatory decisions. We recommend further research on the interplay between provider launches, delivery workforce, and end-customer reactions to innovative delivery services. *Second*, we chart the dependence between different automation technologies, but more investigation is needed to examine the role of varied delivery solutions such as safe lockers, trunk-delivery, and other novel arrangements. Public acceptance is likely to evolve alongside advancements in technology and the modernization of parcel drop-off options. Whereas this work describes realistic drop-off locations as attributes of different delivery options, future work should explicitly investigate the impact of drop-off location options and attitudes concerning package safety, especially considering changing e-commerce and work-from-home patterns^51^. *Third*, while this study shines some light on the impact of the delivered item on the choice of delivery alternatives, data on item type is still limited. Future research should therefore delve further into the explicit impact of delivered item types on choice. *Fourth*, the survey distribution method has biases related to self-selection and the online distribution channel. *Fifth*, any choice experiment is subject to hypothetical bias whereas modeled choices may not match decisions that customers would make in the real world. Recent evidence suggests that while choices might effectively translate to real-world situations, the associated level of certainty does not^52^. Thereby, dual-response choice experiments, such as a choice exercise that not only asks respondents to choose an alternative but also to state the certainty in the choice, might yield more nuanced findings regarding the future of automated delivery.

While these issues may limit the generalizability of our findings, this study provides new insight into the behavioral intentions to accept automated delivery solutions and the dependencies among automation options, while also filling a gap concerning the role of delivery attitudes, items, and attributes.

## Methods

### Survey administration and demographics

The results presented in this study are based on a Qualtrics-designed web survey with a choice experiment administered to 700 contiguous U.S. respondents via Prolific, an online subject recruitment platform. Eight responses are removed due to being low-quality (straight-lining, extreme hastiness, etc.). Table 1 provides an overview of key sample statistics along with the response counts from the four states with the highest representation. The final survey instrument was approved by the Institutional Review Board at Northwestern university (STU00212452). Sample statistics for the final dataset of 692 respondents are shown in Table 1. Looking at the age distribution, the sample leans towards adults under the age of 45, with a mean and median age of 33.4 and 30 years respectively. The average and the median income for the sample are $72,100 and $62,500 annually, compared to $88,600 and $62,800 in the 2019 5-year American Community Survey^53^.

**Table 1:** Sample statistics.

Statistics	Sample (responses)	Sample (%)
State^a^		
California	110	15.9
Texas	55	7.9
New York	52	7.5
Florida	46	6.6
Gender		
Male	346	50.0
Female	332	48.0
Non-binary	14	2.0
Age		
18–24 years	207	29.9
25–34 years	230	33.2
35–44 years	136	19.7
45–54 years	56	8.1
55–64 years	39	5.6
65 years or older	24	3.5
Occupation		
Employed	380	54.9
Student	126	18.2
Other (retired, unemployed, etc.)	186	26.9
Highest level of education		
Middle school or no education	3	0.4
High school	175	25.3
Undergraduate or trade school	399	57.7
Graduate	115	16.6
Household location		
Urban	198	28.6
Suburban	402	58.1
Rural	92	13.3
Race/ethnicity		
White	466	67.3
Asian	112	16.2
Black	56	8.1
Hispanic or Latino	46	6.6
Other	12	1.7
Income^b^		
< $25,000	112	16.6
$25,000–$49,999	156	23.1
$50,000–$99,999	255	37.8
$100,000–$149,999	97	14.4
≥ $150,000	55	8.2

^a^Responses are from 46 out of the 48 contiguous states and Washington, D.C. The two states not represented in the data, Vermont and Wyoming, are the least populated U.S. states, each accounting for 0.2% of the total US population.

^b^Non-responses for income: 17.

### Delivery automation choice experiment design

The main section of the survey is a discrete choice experiment (DCE) designed to identify the main factors that drive customer preferences for automated modes of parcel delivery in the context of different delivery attributes and shipment items. For the experiment, participants are asked to choose their preferred shipping option for home delivery of an item purchased via e-commerce. Prior to the choice scenarios, respondents are provided with detailed descriptions of the different automated technologies. Considering the novelty of these technologies, the descriptions assume no prior knowledge on the respondents’ behalf. The descriptions are carefully curated to use neutral language designed to not bias opinions of these modes. For additional clarity, each of the descriptions is accompanied by an image of the respective delivery mode.

Respondents are presented with 6 hypothetical choice scenarios with delivery options for three different item types. The labeled choice experiment includes five alternatives, a status quo traditional truck option, autonomous vehicles, aerial drones, sidewalk robots, and bipedal robots. Four shipping attributes are included in the choice experiment, refined via a review of the literature and through a pilot survey. These are: (1) shipping cost, (2) shipping time, (3) recipient’s presence required at delivery, (4) flexible delivery option allowing the recipient to choose a 2-h delivery window (see Supplementary Fig. S2 for a sample choice scenario from the survey). While not an explicit feature within the choice experiment, each of the modes are assigned realistic drop-off locations that are assumed to enter the choice evaluation. Respondents are asked to assume that they do not have any subscription that entitles them to expedited free shipping. Additionally, these scenarios cycle through 3 different types of delivered items (2 scenarios each) to capture nuances in behavior for different shipment categories, targeting item-specific attributes such as *fragility*, *item cost,* and *perishability* (see Supplementary Table S3 for more detail on experimental design factors).

The choice experiment is a D-efficient experimental design generated and evaluated in *Ngene 1.2.1*^54^. Prior parameter estimates were obtained from a pilot of 60 respondents. The efficient design is designed to minimize the asymptotic variance–covariance matrix and render lower the standard errors by extracting more information from a given sample size. The methodology focuses on a given treatment repetition, attribute and level combinations, and the context effect (delivered items). Additionally, we added appropriate conditions to avoid dominant alternatives, generating 4.9 million designs of 30 choice scenarios (blocked into 5 sets of 6 tasks) in a span of 22 days (single-threaded on Intel Core i7-6700 @ 3.40 GHz). The most favorable design was ultimately presented to respondents.

### Attitudes and behavioral data

Other sections of the survey capture respondents’ attitudes towards shipping, technology, and the environment through carefully selected indicators. More specifically, the intent is to capture three latent variables: *affinity towards technology, concerns regarding privacy and package handling,* and *environmental consciousness*. Previous work has shown that affinity towards technology and environmental consciousness affect the adoption of automated technologies^20^. The indicators are captured through 5-point Likert scale questions ranging from *strongly disagree (1)*, to *neither agree nor disagree (3)*, to *strongly agree (5)*. The remaining sections of the survey include questions about the respondents, including shopping habits, socioeconomic status, demographics, and the impact of the COVID-19 pandemic on respondents and their households. Attitude statements included in the measurement model are shown in Supplementary Table S5.

## Modeling framework and estimation

### Hybrid choice model

To capture the grouping of similar technologies within the decision-making process and account for latent attitudinal factors, a model framework combining *nested logit* and *hybrid choice* models is used in this work. Nested logit models group alternatives that are similar to one another, specifically with respect to unobserved or excluded characteristics, into different nests^55,56^. In this study, we expect that some modes share characteristics in the decision-making process that are not present in other modes (e.g. automated vs. traditional, ground vs. aerial, etc.). We tested 10 different configurations, covering nested and cross-nested models, as well as multiple levels of nesting (see Supplementary Fig. S1). The structure in Fig. 2 emerged as the preferred structure with the small-scale automated delivery options bundled together.

Using that structure, the utility equations for these alternatives are provided below. The utility equations are expanded to include shared observed components ($V$) and error terms ($\varepsilon$) for grouped alternatives in addition to the distinct alternative-specific elements.

1 $$\begin{aligned}{U}_{traditional}&={V}_{traditional}+{\varepsilon }_{traditional} \\ {U}_{AV}&={V}_{AV}+{\varepsilon }_{AV} \\ {U}_{drone}&={V}_{nonvehicular}+{V}_{drone}+{\varepsilon }_{nonvehicular}+{\varepsilon }_{drone} \\ {U}_{sidewalk robot}&={V}_{nonvehicular}+{V}_{sidewalk robot}+{\varepsilon }_{nonvehicular}+{\varepsilon }_{sidewalk robot} \\ {U}_{bipedal robot}&={V}_{nonvehicular}+{V}_{bipedal robot}+{\varepsilon }_{nonvehicular}+{\varepsilon }_{bipedal robot} \end{aligned}$$

**Figure 6:**
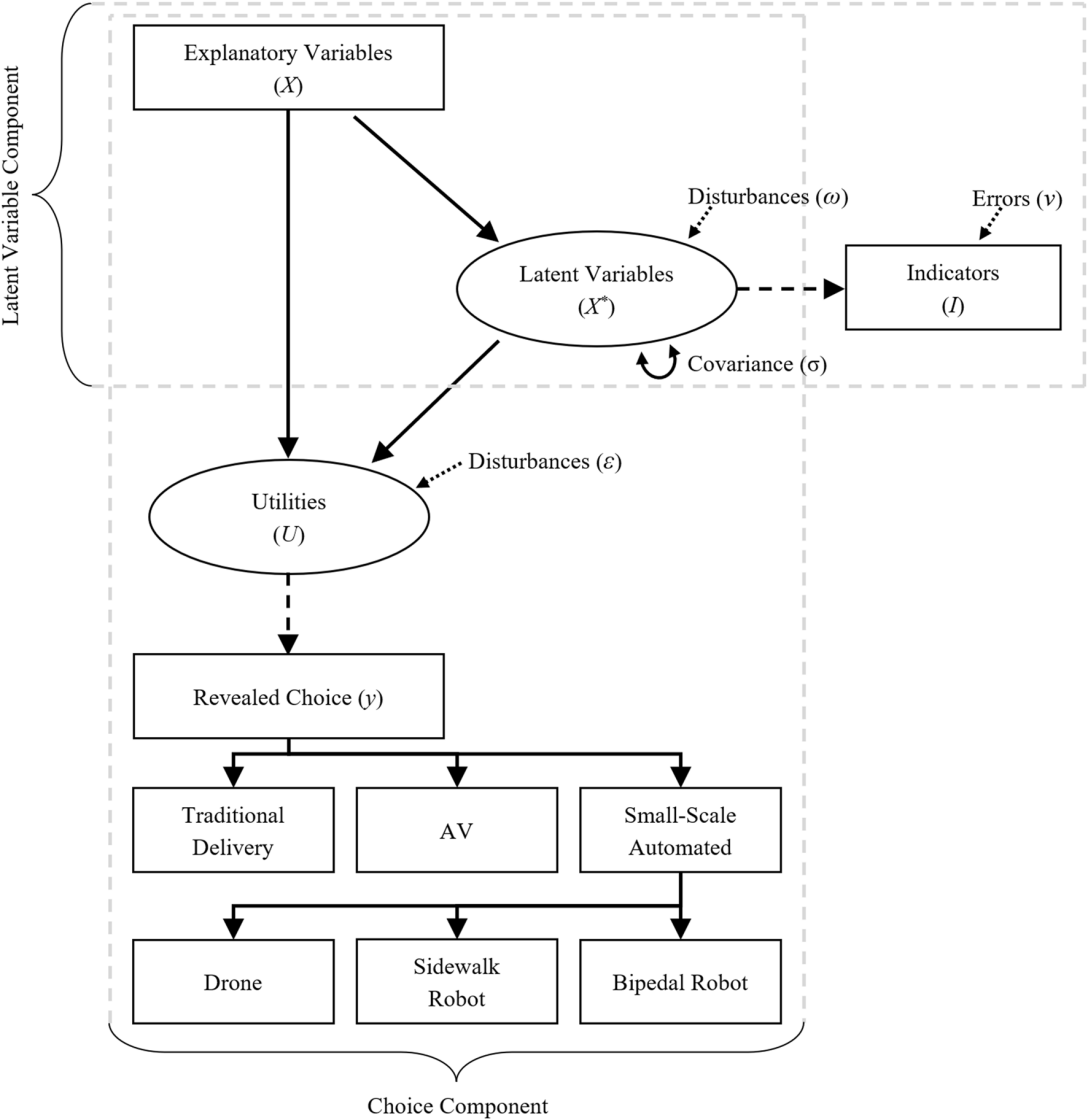
Integrated Nested Choice and Correlated Latent Variable (INCLV) model framework.

Hybrid models (also known as integrated choice and latent variable models) extend choice models by considering the impact of attitudes, perceptions, and psychometric constructs on the decision-making process through the inclusion of a latent variable model^57^. A hybrid choice model consists of a latent variable model, capturing the relationships between explanatory variables and attitudinal indicators with the latent variables, and a choice model component. The resulting full model structure and explanations of terms is shown in Fig. 6^58,59^. The measurement and structural equations forming the latent variable component of the hybrid choice model are given as,

2 $${X}^{*}={X}^{*}\left(X;\alpha ,\omega \right){X}^{*}={X}^{*}\left(X;\alpha ,\omega \right) \; \; \mathrm{where} \; \; \omega \sim D\left(0,{\Sigma }_{\omega }\right)\omega \sim D\left(0,{\Sigma }_{\omega }\right)$$

3 $$I=I\left(X,{X}^{*};\lambda ,\nu \right)I=I\left(X,{X}^{*};\lambda ,\nu \right) \; \; \mathrm{ where} \; \; \nu \sim D\left(0,{\Sigma }_{\nu }\right)\nu \sim D\left(0,{\Sigma }_{\nu }\right)$$

The utility equation is shown below. $\beta$, *α* and *λ* are all parameters to be estimated.

4 $$U=U\left(X,{X}^{*};\beta ,{\varepsilon }_{n}\right)U=U\left(X,{X}^{*};\beta ,{\varepsilon }_{n}\right)\; \; \mathrm{where} \; \;\varepsilon \sim D\left(0,{\Sigma }_{\varepsilon }\right)\varepsilon \sim D\left(0,{\Sigma }_{\varepsilon }\right)$$

### Correlated latent variables

To better capture the relationship between latent variables and their influence on one another, the model is further extended to allow for correlations among the three latent constructs by using Cholesky decomposition to decompose the variance–covariance matrix of the latent variables (see Supplementary Material for the derivation). For a more detailed treatment of nested models and hybrid choice models, the reader is referred to Washington et al.^56^ and Abou-Zeid and Ben-Akiva^57^, respectively.

### Estimation parameter sensitivity

The INCLV model presented in this study is estimated using *apollo v0.2.7* package^60^ in *R v 4.1.2*^61,62^. Specifically, the estimation process uses 20,000 antithetic scrambled Sobol draws, converging in 17 h and 57 min on an Intel Xeon Gold 6230 CPU (28 cores at 2.10 GHz). The high number of draws in conjunction with Sobol draws improves precision and reduces simulation bias^63^. Given the model complexity, we completed parameter sensitivity testing to verify the stability of the final parameters. The results suggest that estimates are reliable based on model stability testing with 20 randomly selected parameter starting values (with 500 Sobol draws; see Supplementary Fig. S3 for sensitivity testing results and comparison to the final model).

### Supplementary Information

Supplementary Information.

## Data Availability

The authors are committed to promoting transparency and open science. Reasonable requests for access to an anonymized version of the dataset can be made by contacting the corresponding author.
