# Supplementary material for: Robots at your doorstep: acceptance of near-future technologies for automated parcel delivery
Source: Sci Rep. 2023 Oct 29;13:18556. doi: 10.1038/s41598-023-45371-1 (PMC10613628; doi:10.1038/s41598-023-45371-1)
Supplement: Supplementary file 1 — Supplementary Information. [file 41598_2023_45371_MOESM1_ESM.pdf]

SUPPLEMENTARY MATERIAL

Model Results

Table S1 INCLV Model metrics and statistics

Metric/Statistic	Value
Number of respondents	692
Data points	692 × 6 = 4,152
Draws type	Scrambled Sobol (antithetic)
Number of draws	20,000
Null log-likelihood	-17,482
Final log-likelihood	-15,329
Adj. McFadden's ρ^2	0.121
AIC	30,747
BIC	31,079

Table S2 Results of INCLV model for novel delivery modes

Parameter	Mode	Coefficient	t-score	p-value
**Alternative-Specific Constants**	Autonomous Vehicle (AV)	1.115 ***	4.854	0.000
	Delivery Drone	1.320 ***	5.140	0.000
	Sidewalk Robot	1.213 ***	4.983	0.000
	Bipedal Robot	0.784 ***	4.181	0.000
**Delivery Cost/Income ($/$10,000)**	Traditional Delivery †	-0.459 ***	-7.693	0.000
	Autonomous Vehicle †	-0.459 ***	-7.693	0.000
	Delivery Drone	-0.548 ***	-5.705	0.000
	Sidewalk Robot †	-0.797 ***	-5.674	0.000
	Bipedal Robot †	-0.797 ***	-5.674	0.000
**Delivery Cost ($)** *(missing income only)*	All modes	-0.333 ***	-4.635	0.000
**Delivery Time (days)**	Traditional Delivery	-0.089 ***	-4.395	0.000
	Autonomous Vehicle	-0.343 ***	-16.396	0.000
	Delivery Drone	-0.248 ***	-7.112	0.000
	Sidewalk Robot †	-0.159 ***	-6.838	0.000
	Bipedal Robot †	-0.159 ***	-6.838	0.000
**Recipient Presence Required**	Traditional Delivery	n.s.	n.s.	n.s.
	Autonomous Vehicle	0.575 ***	5.564	0.000
	Delivery Drone	0.260 **	3.256	0.001
	Sidewalk Robot	-1.239 ***	-5.099	0.000
	Bipedal Robot	-0.441 ***	-4.682	0.000
**Presence Required × Flexible Delivery Window**	Traditional Delivery	n.s.	n.s.	n.s.
	Autonomous Vehicle	n.s.	n.s.	n.s.
	Delivery Drone	n.s.	n.s.	n.s.
	Sidewalk Robot	1.036 ***	4.589	0.000
	Bipedal Robot	0.293 **	2.610	0.009
**Delivered Item: Smartphone**	Autonomous Vehicle †	-1.047 ***	-9.928	0.000
	Delivery Drone	-1.696 ***	-9.680	0.000
	Sidewalk Robot †	-1.047 ***	-9.928	0.000
	Bipedal Robot †	-1.047 ***	-9.928	0.000
**Delivered Item: Groceries**	Autonomous Vehicle	0.633 ***	5.963	0.000
	Delivery Drone †	-0.476 ***	-5.016	0.000
	Sidewalk Robot	n.s.	n.s.	n.s.
	Bipedal Robot †	-0.476 ***	-5.016	0.000
**Age (years)**	Autonomous Vehicle	-0.194 ***	-4.555	0.000
	Delivery Drone	-0.083 *	-2.237	0.025
	Sidewalk Robot	-0.194 ***	-4.555	0.000
	Bipedal Robot	n.s.	n.s.	n.s.
**Gender: Female**	Automated only	-0.273	-1.895	0.058
**Ethnicity: Asian or Pacific Islander**	Automated only	0.457 *	2.560	0.010
**Education: Graduate**	Automated only	0.336	1.905	0.057
**Affinity Towards Technology**	Automated only	2.152 ***	6.440	0.000
**Environmental Consciousness**	Automated only	-0.510	-1.264	0.206
**Privacy & Handling Concerns**	Automated only	-1.533 ***	-4.457	0.000
**Latent Variable Covariances**	*Affinity Towards Technology - Environmental Consciousness*	0.434 ***	4.292	0.000
	Affinity Towards Technology - Privacy & Handling Concerns	0.654 ***	6.658	0.000
	Environmental Consciousness - Privacy & Handling Concerns	0.256 *	2.193	0.028
**Nesting Parameter (μ)**	Small-scale automated modes	0.693 ***	6.948	0.000
	(t-test for μ=1)		-3.080	0.002

*** = significant at 0.001 level; ** = significant at 0.01 level; * = significant at 0.05 level; † indicates that a coefficient is fixed to another with a generalized parameter

Table S3 Choice experiment design Mode attribute levels, drop-off locations and item type attributes

Modes and delivery locations							
Autonomous vehicle	nearest curbside facing delivery address						
Drone	nearest outdoor drop-off location (building entrance or in a front yard)						
Sidewalk robot	nearest outdoor drop-off location (building entrance or in a front yard)						
Bipedal robot	doorsteps or secure locations						
Traditional delivery	doorsteps or secure locations						
Attribute Levels (for all modes)							
Shipping cost	$0 / $3 / $6						
Shipping time	2 days / 5 days / 8 days						
Presence required	No / Yes						
Flexible delivery	No / Yes						
Delivered item type	Cost	Size	Weight	Expensive	Fragile	Perishable	Tamper Risk
Book	$15	small	light	✗	✗	✗	✗
Groceries	$30	medium	medium	✗	✓	✓	✓
Smartphone	$400	small	light	✓	✓	✗	✗

Table S4 Attribute inputs for simulated scenarios

**Attribute, Mode**	**Baseline**	**2-day delivery subscription**	**AV boost**	**Drone boost**	**Sidewalk robot boost**	**Bipedal robot boost**	**Automation boost**	**Supply chain crisis**	**Shipping cost inflation**	**Safe lockbox provision**
**Cost ($)**										
Traditional	8	0	5	5	5	5	8	10	9.6	8
AV	8	0	3	8	8	8	4	7	9.6	8
Drone	8	0	8	3	8	8	4	7	9.6	8
SW Robot	8	0	5	5	3	5	4	7	9.6	8
BP Robot	8	0	8	8	8	3	4	7	9.6	8
**Time (days)**										
Traditional	4	2	2	2	2	2	4	8	4	4
AV	4	2	1	2	2	2	2	5	4	4
Drone	4	2	2	1	2	2	2	5	4	4
SW Robot	4	2	2	2	1	2	2	5	4	4
BP Robot	4	2	2	2	2	1	2	5	4	4
**Flexible Delivery***										
Traditional	0	0	0	0	0	0	0	0	0	0
AV	0	0	0	0	0	0	1	0	0	1
Drone	0	0	0	0	0	0	1	0	0	1
SW Robot	0	0	0	0	0	0	1	0	0	1
BP Robot	0	0	0	0	0	0	1	0	0	1
**Presence Required***										
Traditional	0	0	0	0	0	0	0	0	0	0
AV	1	1	1	1	1	1	1	1	1	0
Drone	0	0	0	0	0	0	0	0	0	0
SW Robot	1	1	1	1	1	1	1	1	1	0
BP Robot	0	0	0	0	0	0	0	0	1	0

*1 = "yes" (flexible delivery window is an option/presence is required); 0 = "no"

Table S5 Results for latent variable (measurement) component of INCLV model

Indicator	Coefficient	t-score	p-value
**Affinity towards technology**			
Technology is changing society for the better.	0.534 ***	11.56	0.000
I am excited to learn about new technologies in the market.	0.622 ***	13.77	0.000
I pay more to get more technologically advanced products.	0.534 ***	13.50	0.000
I use the internet daily for chatting and entertainment.	0.339 ***	6.434	0.000
**Concerns regarding privacy and package handling**			
Technology is changing society for the better.	0.482 ***	9.534	0.000
I am excited to learn about new technologies in the market.	0.456 ***	8.095	0.000
I pay more to get more technologically advanced products.	0.510 ***	9.944	0.000
**Environmental consciousness**			
I am willing to switch to an active mode of transportation (such as walking or cycling) in order to protect the environment.	0.575 ***	15.82	0.000
I would select more environmentally friendly package delivery options at the cost of slower delivery.	0.599 ***	16.62	0.000
I prefer to order items online in bulk to minimize the total number of delivery trips made to my address.	0.412 ***	9.188	0.000
I am concerned with the news about climate change.	0.566 ***	12.29	0.000

*** = significant at 0.001 level; ** = significant at 0.01 level; * = significant at 0.05 level; † indicates that a coefficient is fixed to another with a generalized parameter

Tested Nesting Structures

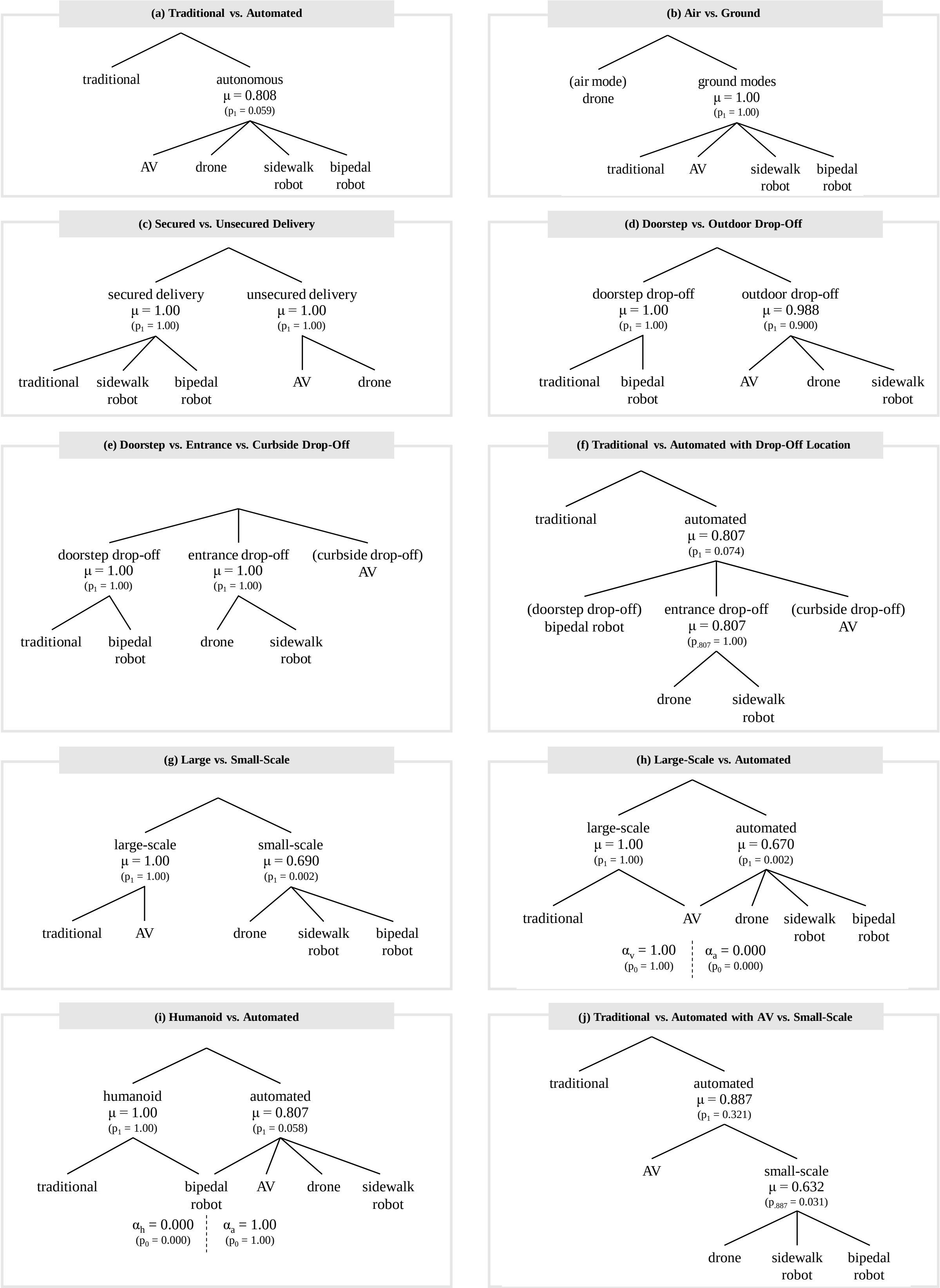

Figure S1 Nesting structures tested during model development. Structure g was ultimately selected

Imagine that you are buying a **book that costs $15**. Upon purchase, you are provided with the following shipping options.

Delivery Method	Traditional Delivery	Autonomous Vehicle	Drone	Sidewalk Robot	Bipedal Robot
**Shipping Cost**	$6	$3	$0	$3	$6
**Shipping Time**	2 days	2 days	8 days	8 days	2 days
**Presence Required?**	No	No	Yes	Yes	Yes
**Flexible Delivery?**	Yes	No	No	Yes	Yes

Imagine that you have the five different delivery options to deliver your purchase.
Which delivery option would you select?

- ○ Traditional Vehicle
- ○ Autonomous Vehicle
- ○ Drone
- ○ Sidewalk Robot
- ○ Bipedal Robot

Figure S2 Sample choice scenario from the survey

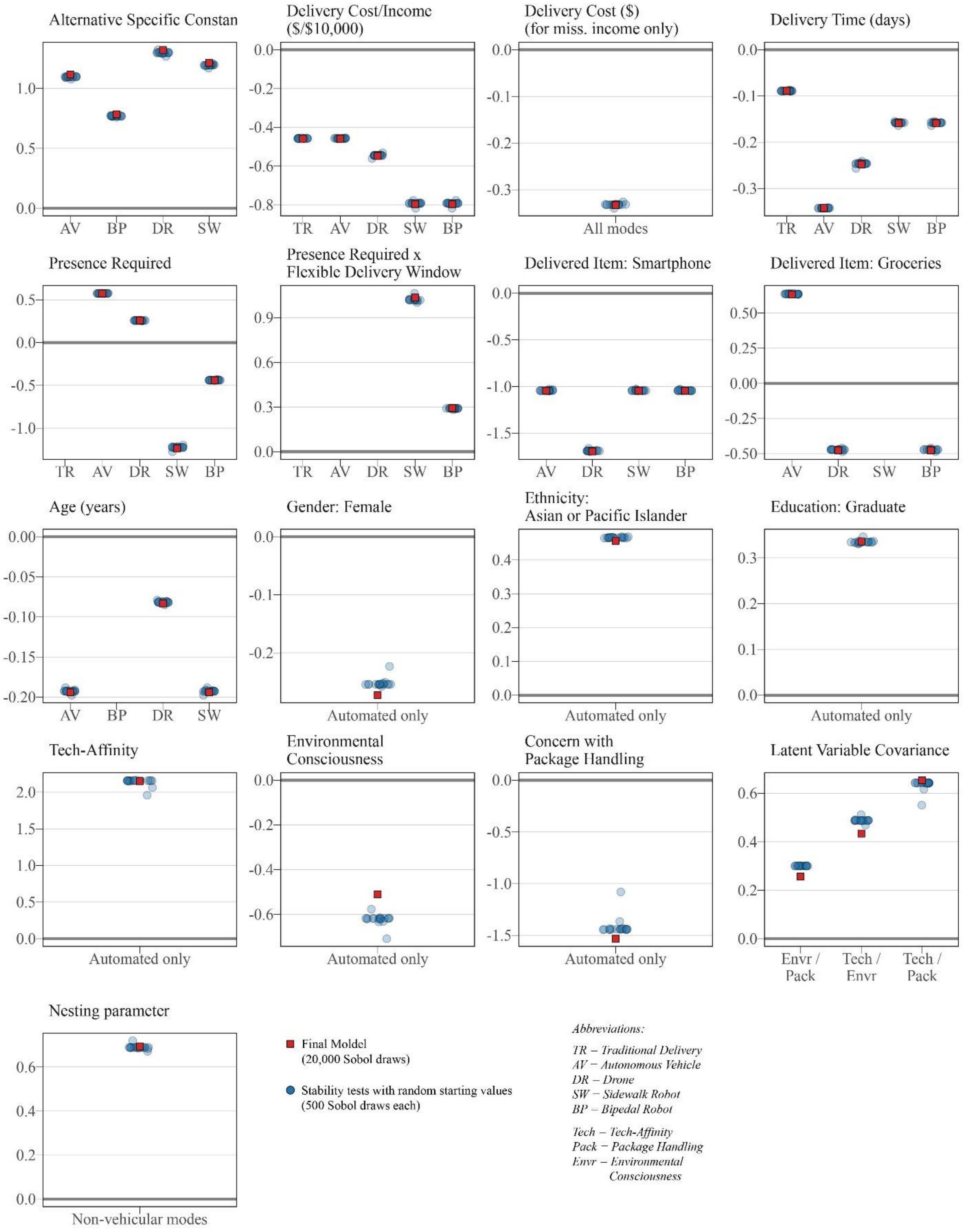

Figure S3 Stability testing for estimated choice model

Capturing Latent Variable Correlation using Cholesky Decomposition

Let Σ_{X^*} be the variance-covariance matrix for the latent variables and L be a lower triangle matrix of variables to be estimated. Accordingly, the Cholesky decomposition is formulated as follows.

$$\Sigma_{X^*} = LL^T \tag{1}$$

In a system of 3 latent variables, the decomposition is expanded below. Variance terms are fixed to 1 for identification.

$$\begin{pmatrix} 1 & \sigma_{12} & \sigma_{13} \\ \sigma_{21} & 1 & \sigma_{23} \\ \sigma_{31} & \sigma_{32} & 1 \end{pmatrix} = \begin{pmatrix} a & 0 & 0 \\ b & c & 0 \\ d & e & f \end{pmatrix} \begin{pmatrix} a & b & d \\ 0 & c & e \\ 0 & 0 & f \end{pmatrix} \tag{2}$$

The terms a to f are solved for using the Cholesky-Banachiewicz algorithm. The resulting matrix L is then multiplied by a vector Ψ of random variates, yielding the following.

$$L\psi = \begin{pmatrix} \psi_1 & 0 & 0 \\ \psi_1\sigma_{12} & \psi_2\sqrt{1-\sigma_{12}^2} & 0 \\ \psi_1\sigma_{13} & \psi_2\dfrac{\sigma_{23}-\sigma_{12}\sigma_{13}}{\sqrt{1-\sigma_{12}^2}} & \psi_3\sqrt{1-\sigma_{13}^2-\dfrac{(\sigma_{23}-\sigma_{12}\sigma_{13})^2}{1-\sigma_{12}^2}} \end{pmatrix} \tag{3}$$

The variance-covariance matrix is then estimated by adding the terms a to f to the structural equations of the model's latent variable component.
